# Connectivity changes in major depressive disorder after rTMS: a review of functional and structural connectivity data

**DOI:** 10.1017/S2045796021000482

**Published:** 2021-09-06

**Authors:** G. Schiena, G. Franco, A. Boscutti, G. Delvecchio, E. Maggioni, P. Brambilla

**Affiliations:** 1 Department of Neurosciences and Mental Health, Fondazione IRCCS Ca’ Granda Ospedale Maggiore Policlinico, Milan, Italy; 2 Department of Pathophysiology and Transplantation, University of Milan, Milan, Italy

**Keywords:** Connectivity, depression, neuromodulation, repetitive transcranial magnetic stimulation

## Abstract

**Aims:**

In the search for effective therapeutic strategies for depression, repetitive transcranial magnetic stimulation (rTMS) emerged as a non-invasive, promising treatment. This is because the antidepressant effect of rTMS might be related to neuronal plasticity mechanisms possibly reverting connectivity alterations often observed in depression. Therefore, in this review, we aimed at providing an overview of the findings reported by studies investigating functional and structural connectivity changes after rTMS in depression.

**Methods:**

A bibliographic search was conducted on PubMed, including studies that used unilateral, excitatory (⩾10 Hz) rTMS treatment targeted on the left dorsolateral prefrontal cortex (DLPFC) in unipolar depressed patients.

**Results:**

The majority of the results showed significant TMS-induced changes in functional connectivity (FC) between areas important for emotion regulation, including the DLPFC and the subgenual anterior cingulate cortex, and among regions that are part of the major resting-state networks, such as the Default Mode Network, the Salience Networks and the Central Executive Network. Finally, in diffusion tensor imaging studies, it has been reported that rTMS appeared to increase fractional anisotropy in the frontal lobe.

**Limitations:**

The small sample size, the heterogeneity of the rTMS stimulation parameters, the concomitant use of psychotropic drugs might have limited the generalisability of the results.

**Conclusions:**

Overall, rTMS treatment induces structural and FC changes in brain regions and networks implicated in the pathogenesis of unipolar depression. However, whether these changes underlie the antidepressant effect of rTMS still needs to be clarified.

## Introduction and aims

Depressive disorders are one of the leading causes of disability worldwide, with a high impact on individuals and society in terms of medical costs and loss of productivity (Friedrich *et al.,* 2017). Major depressive disorder (MDD) is one of the most common mental disorders, with an estimated worldwide prevalence rate of 4.7% (Friedrich, 2017). Notably, approximately 15–30% of MDD patients do not respond to two antidepressant drugs, defining the condition as treatment-resistant depression (TRD), which is associated with more severe cognitive impairment, increased comorbidities, increased risk of suicide and higher medical costs (Du *et al*., 2017; Garay *et al*., 2017).

Various treatment strategies have been proposed for TRD, including both pharmacological and non-pharmacological approaches (McIntyre *et al*., 2014). Among the latter, various stimulation techniques have been approved for TRD, such as electroconvulsive therapy, vagus nerve stimulation, deep brain stimulation and repetitive transcranial magnetic stimulation (rTMS) (Akhtar *et al*., 2016). Specifically for rTMS, this technique is progressively gaining ground as a non-invasive, safe and generally well-tolerated treatment option for MDD and its efficacy in patients with TRD has been confirmed in three large, multicentre, randomised controlled trials (RCTs) (O'Reardon *et al*., 2007; George *et al*., 2010; Levkovitz *et al*., 2015). Briefly, during rTMS sessions, repeated magnetic pulses are delivered through the skull to a specific cortical region; when the magnetic field reaches the neural tissues, a secondary electrical field is generated. The ultimate effect is dependent on stimulation frequency, with high-frequency rTMS associated with increased neuronal excitability and low-frequency stimulations with decreased neuronal excitability (De Risio *et al*., 2020).

Several mechanisms of action have been postulated for rTMS in the treatment of MDD (Chervyakov *et al*., 2015). A prominent hypothesis suggests that rTMS induces neuronal plasticity and a restructuration of neuronal networks (Kozyrev *et al*., 2018). This is relevant since MDD has been associated not only with structural brain alterations, but also with functional connectivity (FC) dysfunctions of major brain networks (Schmaal *et al*., 2020), including the Central Executive Network (CEN), which is involved in cognitive control and emotion regulation, and the Default Mode Network (DMN), which mediates self-referencing and internally oriented processes (Hamilton *et al*., 2015; Kaiser *et al*., 2015).

In this framework, the aim of this review is to summarise the evidence on brain structural and FC changes after excitatory rTMS of the left dorsolateral prefrontal cortex (DLPFC) in MDD, and their potential relation with rTMS therapeutic efficacy.

## Methods

We performed a bibliographic search on PubMed, with the following query: ‘(rTMS or TMS or transcranial magnetic stimulation) AND (connectivity or dwi or diffusion) AND (depression)’. No limitation was posed regarding publication date. We included studies using unilateral, excitatory, high-frequency (⩾10 Hz) TMS treatment targeting the left DLPFC. Only studies that investigated brain structural and FC using whole-brain neuroimaging techniques before and after rTMS protocols in depressed patients were included. Studies (a) employing other techniques (e.g. electroencephalogram), (b) based on bilateral or inhibitory rTMS protocols, or (c) targeting areas other than left DLPFC were excluded. The reference list of the selected articles was checked in order to find relevant references not emerged from the main query. Only 13 studies were selected as eligible. Of these, two studies included, in addition to unipolar depressed patients, a small cohort of depressed bipolar disorder type II patients. Finally, nine studies used resting-state functional magnetic resonance imaging (rs-fMRI), three studies employed diffusion tensor imaging (DTI) techniques, and one study employed single-photon emission computed tomography (SPECT).

## Results

Connectivity changes induced by rTMS treatment are briefly discussed below, divided according to the investigated connectivity domain. FC results focused on two brain areas, the DLPFC and the subgenual anterior cingulate cortex (sgACC), whose connectivity emerged to be affected by the selected rTMS protocol. Although our focus was on connectivity changes from before to after rTMS, we also listed, when reported, the baseline features predictive of treatment response.

### DLPFC functional connectivity changes

In an open-label trial on 58 unipolar and bipolar depressed patients evaluating SPECT FC changes before and after 4 weeks of rTMS, Richieri *et al*. (2018) demonstrated a decrease in FC between the left DLPFC and both the anterior and posterior cingulate cortex and the right medial temporal lobe, key nodes of the DMN. Similarly, in another open-label trial on 17 unipolar and bipolar type II depressed patients and 35 healthy controls (HC) evaluating rs-fMRI connectivity changes before and after 5 weeks of rTMS, Liston *et al*. (2014) found, at baseline, a decreased FC in patients compared to HC between the left DLPFC and a key region of the DMN, the right parahippocampal gyrus. Moreover, consistently with the results reported by Richieri *et al*. (2018), the authors observed an rTMS-induced decrease in FC between the left DLPFC and many areas of the DMN, such as the ventromedial prefrontal cortex, the posterior cingulate cortex and the right parahippocampal gyrus. Besides the investigation of FC between DLPFC and DMN areas, Liston *et al*. (2014) also explored FC between DLPFC and regions that are part of the CEN. Specifically, the authors showed, at baseline, a decreased FC in patients compared to HC between the left DLPFC and multiple areas of the CEN, including the premotor cortex, inferior parietal lobule, precuneus, cerebellum and other areas within the lateral prefrontal cortex. However, the FC reductions in these areas did not change after rTMS.

Moreover, in an open-label trial on 27 unipolar depressed patients and 27 HC, Zheng *et al*. (2020) analysed rs-fMRI connectivity changes before and after 2 weeks of rTMS through functional connectivity density, defined as the FC between a voxel and the rest of voxels across the whole brain. Consistently with the results found by Liston *et al*. (2014), the authors observed, at baseline, a decreased FC in patients compared to HC within the CEN. However, this decreased FC improved after rTMS, contrasting with the null effect of rTMS reported by Liston *et al*. (2014). Additionally, in an RCT on 21 unipolar depressed patients evaluating rs-fMRI connectivity changes after 2 weeks of real *v*. sham rTMS, Kang *et al*. (2016) demonstrated a decreased FC in active compared to sham group between both targeted (left) DLPFC and contralateral DLPFC and between the left DLPFC and left caudate. Consistently with these results, in an RCT on 33 unipolar depressed patients evaluating rs-fMRI connectivity changes before and after 4 weeks of rTMS, Eshel *et al*. (2020) observed a decreased FC in active compared to sham group between both targeted (left) DLPFC and controlateral DLPFC and between the left DLPFC and bilateral amygdala. Moreover, the authors demonstrated an increased targeted (left) DLPFC global FC in active compared to sham group. All these post-rTMS changes brought patients closer to the FC values demonstrated in the HC group. Interestingly, the authors also investigated the modulating role of the left DLPFC stimulation on contralateral DLPFC and bilateral amygdala through the analysis of the fMRI blood oxygen level-dependent signal after the left DLPFC single-pulse TMS. The authors found that, in HC, DLPFC stimulation deactivated bilateral amygdala, causing no change in contralateral DLPFC, whereas in patients it failed to deactivate bilateral amygdala and aberrantly activated contralateral DLPFC. Finally, in an RCT on 27 unipolar depressed patients, Iwabuchi *et al*. (2019) found no difference, after TMS, in FC in the circuit between right amygdala and DLPFC.

### sgACC functional connectivity changes

In the already cited study carried out by Liston *et al*. (2014), the authors also explored the sgACC connectivity. Specifically, the authors demonstrated, at baseline, an increase in FC between sgACC and multiple DMN areas, including the ventromedial prefrontal cortex, pregenual anterior cingulate cortex (pgACC) and precuneus, in patients compared to HC, which reverted after rTMS. A similar reversal of the FC alterations between sgACC and pgACC was demonstrated by Baeken *et al*. (2014), in a study on 20 unipolar depressed patients evaluating rs-fMRI connectivity changes induced by rTMS. Also, Taylor *et al*. (2018), in an RCT on 32 unipolar depressed patients exploring rs-fMRI connectivity changes before and after 4 weeks of real *v*. sham rTMS, investigated FC between sgACC and both DMN and CEN. Specifically for the DMN, they demonstrated, in responders (both to real and sham stimulation), a decrease in FC between sgACC and DMN, consistently with the result reported by Liston *et al*. (2014). Interestingly, no specific effect of rTMS (real *v.* sham) on the Montgomery-Åsberg Depression Rating Scale (MADRS) and FC was demonstrated, suggesting that reduction in FC between sgACC and DMN may parallel the reduction in depressive symptoms, with no specific effect of active *v*. sham rTMS. Moreover, with regards to the CEN, the authors found a decrease in FC between sgACC and CEN. This last result is in contrast with the one reported by Liston *et al*. (2014), who found no difference in FC connectivity between sgACC and CEN before and after rTMS.

Finally, Ge *et al*. (2020), in an open-label study on 50 unipolar depressed patients exploring rs-fMRI connectivity changes before and after 4–6 weeks of rTMS, demonstrated, at follow-up, a decrease in FC between sgACC and DLPFC, fusiform gyrus and middle occipital cortex, both in responders and in non-responders.

### Structural connectivity changes in white matter tracts

Three studies examined the effect of rTMS on fractional anisotropy (FA), a marker of white matter microstructure, measured through DTI (Kozel *et al*., 2011; Peng *et al*., 2012; Tateishi *et al*., 2019). All these studies included unipolar depressed patients. While Tateishi *et al*. (2019) did not use a control group, the other two studies employed a sham stimulation group (Kozel *et al*., 2011; Peng *et al*., 2012) and a group of HC (Peng *et al*., 2012). Specifically, Peng *et al*. (2012) observed that unipolar depressed patients showed, at baseline, decreased FA in the left middle frontal gyrus compared to HC. After real rTMS, the authors also found increased FA in the left middle frontal gyrus whereas Tateishi *et al*. (2019) found higher FA values in the right superior frontal gyrus. Interestingly, Tateishi *et al*. (2019) observed increased FA only in rTMS non-responders. In contrast, Peng *et al*. (2012) found higher FA changes to be correlated with more pronounced improvement in depressive symptomatology.

Finally, in the sample studied by Kozel *et al*. (2011), no significant difference was found in prefrontal FA values between active and sham rTMS treatment. Indeed, the authors found an FA increase in the left prefrontal white matter only at a trend-level significance.

### Baseline features associated with treatment response

Stronger baseline FC between sgACC and multiple areas of the DMN and the CEN was found to be positively associated with rTMS response (Liston *et al*., 2014). In contrast, weaker baseline FC between the left DLPFC and cingulate cortex, medial frontal cortex and bilateral medial temporal limbic areas (Richieri *et al*., 2018) as well as between the bilateral DLPFC and left caudate was found to be positively associated with rTMS response (Kang *et al*., 2016). Another area whose baseline connectivity was reported to be associated with rTMS response was the right anterior insula (rAI). Specifically, stronger baseline FC between rAI and both DLPFC (Iwabuchi *et al*., 2019) and posterior cingulate cortex (Taylor *et al*., 2018) was found to be positively associated with rTMS response.

## Discussion

In this review, we summarised the results of studies investigating structural and FC changes after excitatory rTMS on the left DLPFC. Interestingly, the results showed that FC changes in key areas involved in the emotion regulation (i.e. DLPFC and sgACC) and major resting-state networks (DMN, CEN, Salience Network (SN)) were found to be associated with rTMS treatment, possibly mediating rTMS therapeutic efficacy.

In particular, in the reviewed studies, a decrease in FC between DLPFC (Liston *et al*., 2014; Richieri *et al*., 2018) or sgACC (Baeken *et al*., 2014; Liston *et al*., 2014; Taylor *et al*., 2018) and DMN areas consistently emerged. Also, stronger baseline rAI connectivity was consistently found to be positively associated with rTMS response, both with DLPFC (Iwabuchi *et al*., 2019) and with the posterior cingulate cortex (Taylor *et al*., 2018).

### Decreased FC between sgACC and DMN areas

The results on sgACC showed that after rTMS, the increased activity of sgACC (Mayberg *et al*., 2000, 2005) and the increased connectivity between sgACC and multiple areas, in particular within the DMN (Greicius *et al*., 2007), found to be associated with depressive symptomatology, seemed to normalise. Notably, this evidence is in line with previous findings showing a similar decrease in hyperactivation and hyperconnectivity of sgACC after very different therapeutic options, such as deep brain stimulation (Mayberg *et al*., 2005), electroconvulsive therapy (Argyelan *et al*., 2016), vagus nerve stimulation (Nahas *et al*., 2007), rTMS targeting the bilateral excitatory dorsomedial prefrontal cortex (Salomons *et al*., 2014), inhibitory right DLPFC rTMS (Kito *et al*., 2008), antidepressant drugs (Mayberg *et al*., 2000; Drevets *et al*., 2002) and the administration of placebo pills, which can result in a clinical response very similar to the one of antidepressant therapies (Mayberg *et al*., 2002). Interestingly, the latter therapeutic option could have occurred in the study performed by Taylor *et al*. (2018) since the decreased FC between sgACC and the DMN and its association with the amelioration of depressive symptomatology reported by this study in the sham group probably suggests a placebo effect of the sham rTMS.

### Decreased FC between DLPFC and DMN areas

The DMN is a network of brain areas active when attention is not focused on the outside world, but is engaged in self-referential processing (Gusnard *et al*., 2001; Raichle *et al*., 2001). This network, which comprises the medial prefrontal, medial posterior parietal cortex and posterior cingulate cortex, has been repeatedly associated with rumination (Zhou *et al*., 2020) and (meta)cognitive style in depression (Gusnard *et al*., 2001; Raichle *et al*., 2001). Indeed, hyperconnectivity within the DMN has been consistently reported by FC studies in MDD patients (for a review, see Kaiser *et al*., 2015). Moreover, when attention is focused on the outside world, the FC between DLPFC and DMN decreases (Piccoli *et al*., 2015; Denkova *et al*., 2019; Bauer *et al*., 2020) and, therefore, the decrease in FC between DLPFC and DMN areas observed in the reviewed studies after rTMS treatment in depressed patients could have facilitated the attention towards the outside world, thus avoiding rumination and improving depressive symptomatology.

### Baseline connectivity

Concerning baseline features associated with treatment response, an association between stronger baseline rAI connectivity and rTMS response consistently emerged from the reviewed studies.

Anterior insula (AI) is part of the SN, a key circuit directing attention and cognitive control (Menon, 2015), and comprising not only the AI but also the dorsal anterior cingulate cortex, amygdala, ventral striatum and substantia nigra/ventral tegmental area. Specifically, AI, and especially the rAI, is crucial to detect and select salient stimuli as well as to interact with other neurocognitive systems, including the DMN and the CEN, by activating or deactivating them according to circumstances (Menon, 2015). Notably, this structure has been often found impaired in depressive disorders (Grimm *et al*., 2009; Sheline *et al*., 2009). Therefore, the stronger baseline FC between rAI and selective areas within the CEN (i.e. DLPFC) and within the DMN (i.e. posterior cingulate cortex), which was consistently found to be positively associated with rTMS response (Taylor *et al*., 2018; Iwabuchi *et al*., 2019), could point towards the hypothesis that rTMS treatment improves the communication between the rAl and these neurocognitive systems, which in turn may have positive effects on depressive symptomatology.

### Structural connectivity changes

The three DTI studies here reviewed (Kozel *et al*., 2011; Peng *et al*., 2012; Tateishi *et al*., 2019) reported increased FA, which suggests an improvement of white matter tracts integrity, in regions within the prefrontal lobe after rTMS treatment. These findings suggest the presence of a normalizing effect of rTMS treatment on prefrontal tracts, which have been often found to be characterised by reduced FA in MDD (Korgaonkar *et al*., 2011; Chen *et al*., 2016), similarly to what has been found for antidepressant treatments (Zeng *et al*., 2012; Gryglewski *et al*., 2020). Therefore, these studies support the hypothesis of a relationship between white matter abnormalities and depressive symptomatology (Walther *et al*., 2012; Coloigner *et al*., 2019; Heij *et al*., 2019), although a clear relationship between white matter deficits and MDD is currently lacking, mainly due to the heterogeneities observed between the studies (Coloigner *et al*., 2019). This is true also for the results reported by the three reviewed studies, since increased prefrontal FA was observed both in the left (Kozel *et al*., 2011; Peng *et al*., 2012) and in the right (Tateishi *et al*., 2019) sides. Therefore, these contrasting results warrant the need for future studies to better clarify the relationship between rTMS treatment and structural connectivity changes in MDD.

## Limitations and conclusions

The reviewed studies suffer from some limitations. First, the sample size was often modest and some studies (Liston *et al*., 2014; Richieri *et al*., 2018) also included a mixed sample of unipolar and bipolar depressed patients, possibly decreasing the statistical power of the statistical analyses. Second, the majority of patients were concomitantly treated with medications, so the observed connectivity changes could be linked to concomitant psychotropic drugs, or placebo effects in open-label studies. Third, the stimulation parameters of rTMS were heterogeneous in terms of TMS frequency, number of sessions, timing and concomitant treatments, possibly influencing the connectivity changes observed.

In conclusion, the abovementioned results support the hypothesis that rTMS induces neuronal plasticity and reorganisation of key networks in the pathogenesis of unipolar depression. However, whether these changes underlie the antidepressant effect of rTMS is not defined yet. Further studies including larger and more homogeneous samples are needed to better clarify the effect of rTMS on brain connectivity and the relationship with its therapeutic effect in unipolar depression.

## Data

All data described in this review have been included in Table 1.

**Table 1.** Connectivity changes in major depressive disorder after rTMS: a review of functional and structural connectivity data

Author	Study design	Sample characteristics	Stimulation parameters	Imaging parameters	Statistical analyses	Main results
Baeken *et al*. (2014)	**Sham-controlled:** Yes (cross-over after 1 week) **Blinded:** Single-blind **Control group:** No **Psychometric assessments:** HAMD17 at baseline, 1 and 2 weeks fup **Neuroimaging assessment:** fMRI at baseline, 1 and 2 weeks fup	**Pts**: 20 (48.8 ± 12.76 years, M/F 7/13) **HC**: NA **Diagnosis**: MDD **Severity**: HAMD 25.65 ± 6.13 **Treatment resistance status:** Thase & Rush stage ⩾ 3; at least 2 failed trials with SSRI/SNRI and 1 with TCA **Medication status:** Washout from all medication 2 weeks before **Psychiatric comorbidities:** Excluded if suicide attempt within previous 6 months, alcohol abuse	**Region:** Left DLPFC **Neuronavigation:** Yes **Intensity (% MT):** 110% **Frequency:** 20 Hz **Protocol:** *Train* = 1.9 s; intertrain interval 12 s *Session* = 40 trains *Total stimuli per session***:** 3000 **Duration:** 4 days/week (5 sessions/day), 1 week	**fMRI (3T)** **Resting state** **FC method:** Seed-based **Regions:** sgACC	**Baseline differences** NA **Post-TMS changes** NA **Relationship with clinical variables** Random-effects two-way ANOVA, with age as covariate, response (positive, negative) as between-subject factor and time (baseline, post-treatment) as within-subject factor Two-sample *t*-tests post hoc for differences in FC between Responders and Non-responders	**Baseline differences** NA **Post-TMS changes** NA **Relationship with clinical variables** At baseline, in Responders *v.* Non-responders increased FC anti-correlation between sgACC and superior medial frontal gyrus and left SFG After rTMS, in Responders *v.* Non-responders inverse correlation effect on FC between sgACC and right pgACC and superior medial frontal gyrus
Chen *et al*. (2020)	**Sham-controlled:** Yes **Blinded:** Double-blind **Control population:** Yes **Psychometric assessments:** HAMD17 at baseline and 4 weeks fup **Neuroimaging assessment:** fMRI at baseline and 12 weeks fup	**Pts**: Active rTMS: 20 (46.75 ± 5.52 years, M/F 9/11) Sham rTMS: 20 (46.30 ± 4.76 years, M/F 12/8) **HC**: NA **Diagnosis**: MDD (DSM-IV) **Severity**: HAMD >20; Active-rTMS 26.95 ± 2.04 Sham-rTMS 25.50 ± 2.01 **Treatment resistance status:** NA **Medication status:** NA **Psychiatric comorbidities:** Excluded if any other Axis-1 diagnosis, high risk of suicide	**Region:** Left DLPFC **Neuronavigation:** No **Intensity (% MT):** 90% **Frequency**: 10 Hz **Protocol:** *Train* = 4 s; intertrain interval 56 s *Session* = 40 trains (2 times/day) *Total stimuli per session*: 1600 (3200/day) **Duration:** 5 days/week, 5 weeks	**FMRI (1.5T)** **Resting state** **FC method:** Seed-based **Regions:** Bilateral amygdalae	**Baseline differences** General mixed linear model analysis for baseline differences in amygdala FC between pts and controls, with age, gender, education level and voxel-wise GM volume map as covariates **Post-TMS changes** General linear mixed model analysis for amygdala FC changes, with age, gender, education level and voxel-wise GM volume map as covariates **Relationship with clinical variables** Correlation analysis and linear regression model to assess relationships between FC changes and symptoms scores changes	**Baseline differences** *Left AN*: In pts *v.* HC, lower FC between amygdala and left INS, right IFG, right SFG, right IPL, right MFG. Higher FC between amygdala and PreCUN *Right AN*: In pts *v.* HC, lower FC between right amygdala and left INS, right IFG, right INS, right IPL, right MFG Higher FC between amygdala and left PreCUN **Post-TMS changes** *Left AN*: In active rTMS arm increased FC between left amygdala and left INS, right IFG, right INS, right IPL *Right AN*: In active rTMS arm increased FC between right amygdala and left INS No changes in the sham-rTMS arm **Relationship with clinical variables** In active rTMS arm, changes in FC between left INS and left amygdala positively correlated with changes in symptoms scores No correlations in the sham-rTMS arm
Eshel *et al*. (2020)	**Sham-controlled:** Yes **Blinded:** Double-blind **Control population:** Yes **Psychometric assessments:** HAMD at baseline and 4 weeks fup **Neuroimaging assessment** fMRI at baseline and 4 weeks fup	**Pts**: Active rTMS 20 (35.0 ± 6.3 years, 10/10 M/F) Sham-rTMS 13 (37.1 ± 11.2, 4/9 M/F) **HC**: 28 (38.9 ± 11.3 years, 15M/13F) **Diagnosis**: MDD (DSM-IV) **Severity**: HAMD: Active rTMS 26.9 ± 7.7 Sham-rTMS 27.0 ± 6.76 **Treatment resistance status:** ⩾1 but ⩽3 failed antidepressant trials **Medication status:** Medication-free or washout from all medication 2 weeks before **Psychiatric comorbidities:** Excluded if psychotic disorder, BD, active SUD	**Region:** Left DLPFC **Neuronavigation:** Yes **Intensity (% MT):** NA **Frequency:** 10 Hz **Protocol:** *Train* = 4 s; intertrain interval 26 s *Session* = 75 trains *Total stimuli per session***:** 3000 **Duration:** 5 days/week, 4 weeks	**fMRI (3T)** **Resting state** **FC method:** Seed-based **Regions:** Left DLPFC	**Baseline differences** NA **Post-TMS changes** Linear mixed models (fixed effects of time, treatment arm × time) for changes in FC in active rTMS *v.* sham rTMS arm. **Relationship with clinical variables** Pearson correlations between changes in FC and changes in symptoms scores Linear mixed models (fixed effects of time, global connectivity and time × connectivity) to assess relationship between baseline FC and degree of symptoms scores changes	**Baseline differences** NA **Post-TMS changes** In active rTMS arm, increase (normalization) in global FC of left DLPFC In the active rTMS arm, decrease (normalization) in global FC of bilateral amygdalae and right DLPFC **Relationship with clinical variables** In the active rTMS arm, positive correlation between degree of global FC increase of left DLPFC and degree of clinical improvement In the active rTMS arm, lower baseline FC of left DLPFC predicts greater clinical improvement
Ge *et al*. (2020)	**Sham-controlled:** No **Blinded:** NA **Control population:** Yes **Psychometric assessments:** HAMD17 at baseline, 4 and 12 weeks fup **Neuroimaging assessment:** fMRI at baseline and 12 weeks fup	**Pts**: Responders 28 (42.25 ± 13.19 years, M/F 11/17), Non-responders 14 (44.07 ± 10.72 years, M7/F7) **HC**: 25 (45.25 ± 12.19 years, M/F 12/12) **Diagnosis**: MDD (DSM-IV) **Severity**: HAMD17 >18. Responders 21.64 ± 4.54 Non-responders 21.86 ± 2.41 **Treatment resistance status:** ATHF stage ⩾1 but ⩽4. ATHF score: Responders 7.21 ± 3.29 Non-responders 8.07 ± 3.79 **Medication status:** NA **Psychiatric comorbidities:** Excluded if actively suicidal, active SUD or any other psychiatric condition	**Region:** Left DLPFC **Neuronavigation:** Yes *Randomized to conventional rTMS or iTBS* *Conventional rTMS* **Intensity (% MT):** 120% **Frequency:** 10 Hz **Protocol:** *Train* = 4 s; intertrain interval 26 s *Session* = 75 trains *Total stimuli per session*: 3000 *Duration*: 5 days/week, 4 weeks *iTBS* **Intensity (% MT):** 80% **Frequency:** 50 Hz (stimuli), 5 Hz (bursts) **Protocol:** *Burst* = group of 3 stimuli *Train* = group of 10 bursts (2 s duration), inter-burst interval 8 s *Run* = group of 20 trains *Session* = 5 runs, with inter-run interval of 5 min *Total stimuli per session*: 3000 **Duration:** days/week, 4 weeks	**fMRI (3T)** **Resting state** **FC method:** Seed-based **Regions:** sgACC, rACC	**Baseline differences** NA **Post-TMS changes** ANCOVA for effect of group, time and group–time interaction on sgACC and rACC FC **Relationship with clinical variables** Pearson correlation between changes in symptoms scores and sgACC and rACC FC after rTMS Regression analyses to assess relationship between baseline FC or changes in FC with degree of symptoms scores improvement (at both 4 and 12 weeks fup) Exploratory path analysis to assess whether baseline sgACC-DLPFC FC or sgACC-DLPFC changes are mediators of the relationship between sgACC-fusiform FC changes and changes in symptoms scores	**Baseline differences** NA **Post-TMS changes** Significant decrease in FC of sgACC-DLPFC, sgACC-fusiform and sgACC-MOC **Baseline relationship with clinical variables** Stronger baseline rACC-IPL FC associated with greater improvement in symptoms score Stronger sgACC-rDLPFC FC associated with smaller improvement in symptoms score sgACC-DLPFC FC AUC for responders/non-responder – remitters/non-remitters classification: 0.87–0.90 rACC-IPL FC AUC for responders/non-responder – remitters/non-remitters classification: 0.75–0.76 **Post-treatment relationship with clinical variables** Smaller decrease of sgACC-left fusiform FC associated with greater improvement in symptoms score **Path analysis** Lower baseline sgACC-DLPFC FC->greater decrease of sgACC-fusiform FC->greater improvement in symptoms score
Iwabuchi *et al*. (2019)	**Sham-controlled:** No **Blinded:** NA **Control population:** No **Psychometric assessments:** BDI, HAMD17 and cognitive assessment at baseline, 4 and 12 weeks fup **Neuroimaging assessment:** fMRI at baseline and 12 weeks fup	**Pts**: 27 (49.85 ± 10.88 years, 15/12 M/F) **HC**: NA **Diagnosis**: MDD (diagnostic criteria NA) **Severity**: HAMD 20.48 ± 7.4 **Treatment resistance status:** Thase & Rush stage ⩾ 1; mean 2.89 ± 1.01 **Medication status:** No changes in the previous 2 weeks No washout **Psychiatric comorbidities:** Excluded if BD or current SUD	**Region:** Left DLPFC, region of greater EC (GC) with right anterior INS **Neuronavigation:** Yes *Randomized to conventional rTMS or iTBS* *rTMS* **Intensity (% MT):** NA **Frequency:** 10 Hz **Protocol:** *Train* = 4 s; intertrain interval 26 s *Session* = 75 trains *Total stimuli per session*: 3000 **Duration:** 4 days/week, 4 weeks *iTBS* **Intensity (% MT):** 80% **Frequency:** 50 Hz (stimuli), 5 Hz (bursts) **Protocol:** *Burst* = group of 3 stimuli *Train* = group of 10 bursts (2 s duration), inter-burst interval 8 s *Run* = group of 20 trains *Session* = 5 runs, with inter-run interval of 5 min *Total stimuli per session*: 3000 **Duration:** 4 days/week, 4 weeks	**fMRI (3T)** **Resting state** **FC method: Seed**-based (right anterior INS)**,** ICA (networks) **EC method:** GC (right anterior INS-DLPFC) **Regions:** Left DLPFC, right anterior INS, DMN, CEN, SN	**Baseline differences** NA **Post-TMS changes** RM-ANOVA with stimulation protocol as covariate (DLPFC, right anterior INS) Randomise permutation-testing (networks) **Relationship with clinical variables** Bivariate Pearson correlation between FC/EC and symptoms scores changes (DLPFC, right anterior INS) *T*-test for FC/EC baseline differences between Responders and Non-responders (DLPFC, right anterior INS) Randomise permutation-testing for FC baseline differences between Responders and Non-responders (networks)	**Baseline differences** NA **Post-TMS changes** No change in FC/EC (DLPFC, right anterior INS) No change in FC (networks) **Relationship with clinical variables** Positive correlation between net right anterior INS outflow to DLPFC at baseline and degree of HAMD score reduction at 1-month fup Net right anterior INS outflow to DLPFC at baseline higher in Responders compared to Non-responders (1-month fup) Positive correlation between SAL FC at baseline and degree of HAMD score reduction at 1-month fup In Responders compared to Non-responders (1-month fup), lower baseline FC between SN and LG, FG and cerebellum
Kang *et al*. (2016)	**Sham-controlled:** Yes **Blinded:** Double-blind **Control population:** No **Psychometric assessments:** HAMD17 and neurocognitive assessment at baseline and 2 weeks fup **Neuroimaging assessment** fMRI at baseline and 2 weeks fup	**Pts**: Active rTMS: 12 (42.8 ± 19.1 years, M/F 3/9) Sham rTMS: 16 (52.2 ± 20.1 years, M/F 1/8) **HC**: NA **Diagnosis**: MDD (DSM-IV) **Severity**: HAMD: Active rTMS 24.1 ± 6.4, Sham rTMS 20.0 ± 4.6 **Treatment resistance status:** At least 1 failed trial with SSRI **Medication status:** No washout **Psychiatric comorbidities:** Excluded if any other psychiatric disorder, high suicide risk, current SUD, past psychotic disorder	**Region:** Left DLPFC **Neuronavigation:** No **Intensity (% MT):** 110% **Frequency:** 10 Hz **Protocol:** *Train* = 5 s; intertrain interval 25 s *Session* = 20 trains *Total stimuli per session*: *1000* **Duration**: 5 days/week, 2 weeks	**fMRI (T: NA)** **Resting state** **FC method:** Seed-based **Regions:** Bilateral DLPFC	**Baseline differences** NA **Post-TMS changes** Two-sample *t*-tests for FC changes differences between active- and sham-rTMS arms Post-hoc analyses for hemispheric effect of seed ROI on differences in FC between active- and sham-rTMS arms **Relationship with clinical variables** Spearman correlation between FC changes and symptoms scores	**Baseline differences** NA **Post-TMS changes** In active *v.* sham rTMS, greater reduction in FC between DLPFC and left caudate **Relationship with clinical variables** Positive correlation between degree of reduction in DLPFC-left caudate FC and degree of improvement in symptoms score Positive correlation between DLPFC-left caudate FC after rTMS and degree of residual symptoms score
Kozel *et al*. (2011)	**Sham-controlled:** Yes **Blinded:** Double-blind **Control population:** No **Psychometric assessments:** MADRS at baseline **Neuroimaging assessment:** MRI at baseline and 4–6 weeks fup	**Pts**: 8 (44.6 ± 10.2, M/F 1/7) **HC**: NA **Diagnosis**: MDD (diagnostic criteria NA) **Severity**: HAMD ⩾ 20 and item 1 score ⩾ 2: Active rTMS 29.2 ± 5.1 Sham rTMS 29.5 ± 2.6 <3 years duration **Treatment resistance status:** Stage ATHF level 2–4 in current episode, or >1 and ⩽3 in a previous episode **Medication status:** All medications (except hypnotics or lorazepam) discontinued after randomization **Psychiatric comorbidities:** Excluded if depression secondary to substances or medical condition, depression with seasonal pattern, SUD within past year, psychotic disorder, BD, OCD, ED, PTSD	**Region:** Left DLPFC **Neuronavigation:** No **Intensity (% MT):** 120% **Frequency:** 10 Hz **Protocol:** *Train* = 4 s; intertrain interval 26 s *Session* = 75 trains *Total stimuli per session*: 3000 **Duration:** 5 days/week, 4–6 weeks	**MRI DTI (3 T)** **Resting state** **FC method:** FA **Regions:** PFC (bilateral)	**Baseline differences** NA **Post-TMS changes** 2 Treatment group (active rTMS, sham rTMS) × 2 region (left PF, right PF) mixed linear model analysis of repeated measures, with baseline FA as a covariate **Relationship with clinical variables** NA	**Baseline differences** NA **Post-TMS changes** Increased FA in left PFC in active *v.* sham rTMS (trend level significance) **Relationship with clinical variables** NA
Liston *et al*. (2014)	**Sham-controlled:** No **Blinded:** NA **Control population:** Yes **Psychometric assessments:** HAMD24 at baseline and 5 weeks fup **Neuroimaging assessment:** fMRI at baseline and 5 weeks fup	**Pts**: 17 (42.3 ± 17.3 years, M/F 3/14) **HC**: 35 (36 ± 16 years, M/F 12/23) **Diagnosis**: 14 MMD, 3 BD II depression (DSM-IV) **Severity**: NA **Treatment resistance status:** At least 2 failed antidepressant trials **Medication status:** No changes in the previous 4 weeks No washout **Psychiatric comorbidities:** Excluded if any other psychiatric disorder, depression with psychotic features, suicidal ideation/behaviour, SUD in the past 3 years	**Region:** Left DLPFC **Neuronavigation:** No **Intensity (% MT):** 120% **Frequency:** 10 Hz **Protocol:** *Train* = 4 s; intertrain interval 26 s *Session* = 75 trains *Total stimuli per session*: 3000 **Duration:** 5 days/week, 5 weeks	**fMRI (3 T)** **Resting state** **FC method:** Seed-based **Regions:** Left DLPFC, sgACC, DMN, CEN. Two FC maps: • Within-network (DLPFC:CEN, sgACC:DMN) • Between-network (DLFPC:DMN, and sgACC:CEN)	**Baseline differences** ANCOVA with age, sex and head movement as covariates **Post-TMS changes** Repeated-measures ANCOVA of pre- *v.* post-TMS FC in pts, with clinical variables and treatment resistance variables as covariates ANCOVA of post-TMS FC in pts *v.* baseline FC in HC, with age, sex and head movement as covariates **Relationship with clinical variables** ANCOVA of baseline FC in Responders *v.* Non-responders, with age, sex, baseline HAM-D score and lifetime number of antidepressant trials as covariates	**Baseline differences** In pts *v.* HC, FC: Within-CEN: Reduced. In particular, reduced FC between left DLPFC and premotor cortex, posterior parietal areas, bilateral cerebellum, lateral PFC Within-DMN: Increased. In particular, increased FC between sgACC and vmPFC, pgACC, thalamus, preCUN sgACC-CEN: Increased FC between sgACC and caudate nucleus and bilateral posterior parietal areas DLPFC-DMN: Reduced FC between DLPFC and right parahippocampal area **Post-TMS changes** Within-DMN: Normalization of FC. Reduction in FC between sgACC and vmPFC, pgACC and preCUN sgACC-CEN: No change in FC (increased FC persisted) DLPFC-DMN: Increased in magnitude of FC reduction between DLPFC and right parahippocampal area. Reduction in FC between DLPFC and vmPFC and PCC
						**Relationship with clinical variables** DLPFC FC at baseline not a significant predictor Increased FC between sgACC and DMN (vmPFC, dmPFC, pgACC, PCC) at baseline associated with better response Increased FC between sgACC and CEN at baseline associated with better response
Peng *et al*. (2012)	**Sham-controlled:** Yes **Blinded:** Double-blind **Control population:** Yes **Psychometric assessments:** BDI and HAMD17 at baseline and 4 weeks fup **Neuroimaging assessment:** MRI at baseline and 4 weeks fup	**Pts**: 30 (26.86 ± 5.27 years, M/F 19/11) **HC**: 25 (28.240 ± 4.980 years, M/F 14/11) **Diagnosis**: MDD (DSM-IV) **Severity**: NA **Treatment resistance status:** At least 2 failed antidepressant trials **Medication status:** Switch to escitalopram 2 weeks before **Psychiatric comorbidities:** Excluded if any psychiatric disorder	**Region:** Left DLPFC **Neuronavigation:** No **Intensity (% MT):** 110% **Frequency:** 15 Hz **Protocol:** *Train* = 4 s; intertrain interval 29 s *Session* = 50 trains *Total stimuli per session*: 3000 **Duration:** 5 days/week, 4 weeks	**MRI DTI (3T)** **FC method:** FA **Regions:** Whole-brain	**Baseline differences** Two-sample *t*-tests on a voxel-by-voxel basis for differences in FA **Post-TMS changes** Two-factor repeated-measures ANOVA for main effects of groups (active *v.* sham), treatment times (pre *v.* post-rTMS) and group × time Two-sample *t*-tests and paired *t*-tests between pre- and post-treatment FA in active and sham groups **Relationship with clinical variables** Pearson correlation between FA and symptoms scores (pre- and post-rTMS)	**Baseline differences** In pts *v.* HC, FA decreased in left MFG **Post-TMS changes** In left MFG, increase in FA only in active rTMS arm. Post-rTMS FA higher in active *v.* sham rTMS arm In right anterior lobe of the cerebellum, increased FA in active-rTMS arm **Relationship with clinical variables** Negative correlations between FA and symptoms scores before and after treatment in both active and sham rTMS arms
Richieri *et al*. (2018)	**Sham-controlled:** No **Blinded:** NA **Control population:** Yes **Psychometric assessments:** BDI, STAI-Y at baseline and 4 weeks fup **Neuroimaging assessment:** SPECT at baseline only	**Pts**: 58 (53.8 ± 14.0 years, M/F 21/37) **HC**: 55 (49.8 ± 16.6 years, M/F 23/32) **Diagnosis**: 44 MDD, 14 BD II depression (DSM-IV) **Severity**: BDI mean 25.9 ± 9.5 Duration 17.3 ± 8.1 months **Treatment resistance status:** At least 2 failed trials with two different antidepressants MSM mean 8.7 ± 2.1 **Medication status:** No changes in the previous 2 weeks No washout **Psychiatric comorbidities:** Excluded if depression with psychotic features	**Region:** Left DLPFC **Neuronavigation:** No **Intensity (% MT):** 120% **Frequency:** 10 Hz **Protocol:** *Train* = 5 s; intertrain interval 25 s *Session* = 60 trains *Total stimuli per session*: 2000 **Duration:** 5 days/week, 4 weeks	**SPECT, 99mTc-ECD** **Resting state** **FC method:** Inter-regional correlation of normalized perfusion values (full factorial model) **Regions:** Left DLPFC	**Baseline differences** Full factorial model of analysis with normalized L DLPFC perfusion values as an interaction covariate to study inter-regional correlation, between patients and HC **Post-TMS changes** NA **Relationship with clinical variables** Full factorial model of analysis with normalized L DLPFC perfusion values as an interaction covariate to study inter-regional correlation, between Responders and Non-responders, with age, gender, BDI and MSM scores as covariates Spearman correlation between FC and symptoms scores in Responders and Non-responders Multiple logistic regression models to classify responder *v.* non-responder on the basis of baseline FC	**Baseline differences** In Responders *v.* HC, higher baseline FC between left DLPFC and right cerebellum **Post-TMS changes** NA **Relationship with clinical variables** In Responders *v.* Non-responders, higher baseline FC between left DLPFC and right cerebellum
Tateishi *et al*. (2019)	**Sham-controlled:** No **Blinded:** NA **Control population:** No **Psychometric assessments:** BDI, HAMD24, WCST, WFT and SCT at baseline and 6 weeks fup **Neuroimaging assessment:** MRI at baseline and 6 weeks fup	**Pts**: 12: (52.8 ± 17.8 years, M/F 5/7) **HC**: NA **Diagnosis**: MDD (DSM-IV) **Severity**: HAMD 20.33 ± 6.87 **Treatment resistance status:** At least 2 failed trials with two different antidepressants **Medication status:** No washout **Psychiatric comorbidities:** Excluded if any other psychiatric disorder, current SUD	**Region:** Left DLPFC **Neuronavigation:** No **Intensity (% MT):** 100% **Frequency:** 10 Hz **Protocol:** *Train* = 4 s; 26 s intertrain interval *Session* = 40 trains *Total stimuli per session*: 1600 **Duration:** 5 days/week, 6 weeks	**MRI DTI (3T)** **Resting state** **FC method:** FA **Regions:** Bilateral SFG and MFG	**Baseline differences** NA **Post-TMS changes** One-sample paired *t*-test for FA changes **Relationship with clinical variables** Spearman correlation between degree of FA changes and symptoms scores changes	**Baseline differences** NA **Post-TMS changes** Increased FA of right SFG **Relationship with clinical variables** In Responders, no significant increase in FA in bilateral SFG and MFG In Non-responders, FA increased in right SFG
Taylor *et al*. (2018)	**Sham-controlled:** Yes **Blinded:** Double-blind **Control population:** No **Psychometric assessment** MADRS, HRSD17, QIDS, GAD7, WSAS, GAF at baseline, weekly and 4 weeks fup **Neuroimaging assessment** fMRI at baseline and 4 weeks fup	**Pts**: Active rTMS 16 (46.9 ± 10.7 years, M/F 5/11) Sham rTMS: 16 (44.13 ± 11.1 years, M/F 6/10) **HC**: NA **Diagnosis**: MDD (DSM-IV) **Severity**: MADRS ⩾ 18: Active rTMS 25.4 ± 5.7 Sham rTMS 21.9 ± 3.1 Duration <5 years **Treatment resistance status:** At least 1 failed antidepressant trial. ATHF current episode: Active rTMS 2.56 ± 1.75 Sham rTMS 2.94 ± 1.77 **Medication status:** No changes in the previous 4 weeks No washout **Psychiatric comorbidities:** Excluded if BD, OCD, PTSD, psychosis, suicidal ideation/behaviour	**Region:** Left DLPFC, region with maximal negative correlation with sgACC **Neuronavigation:** Yes **Intensity (% MT):** 120% **Frequency:** 10 Hz **Protocol:** NA *Total stimuli per session*: 3000 **Duration:** 5 days/week, 4 weeks	**fMRI (3 T)** **Resting state** **FC method:** Seed-based **Regions:** AN: sgACC and bilateral amygdalae FPN: L DLPFC DMN: PCC SN: dACC	**Baseline differences** NA **Post-TMS changes** Seed-to-whole-brain FC: Regression models with baseline MADRS and mean FD change as covariates Seed-to-network FC: ANCOVA for each seed, with seed-to-network values and time as repeated measures, group as a between-subject factor, mean FD change and baseline MADRS as covariates **Relationship with clinical variables** Seed-to-whole-brain FC: Regression models with baseline MADRS and mean FD change as covariates, to assess relationship between symptoms scores change and FC change Seed-to-network FC: ANCOVA for each seed, with seed-to-network values and time as repeated measures, group as a between-subject factor, mean FD change and baseline MADRS as covariates, to assess relationship between symptoms scores change and FC change Seed-to-whole-brain FC: Regression models with baseline MADRS and baseline FD as covariates, to test baseline FC as a predictor of symptoms scores changes Seed-to-network FC: ANCOVA for each seed, with seed-to-network values and time as repeated measures, group as a between-subject factor, baseline FD and baseline MADRS as covariates, to test baseline FC as a predictor of symptoms scores changes	**Baseline differences** NA **Post-TMS changes** No difference between arms in seed-to-whole-brain FC for any of the seeds No difference between arms in seed-to-network FC for any of the networks **Relationship with clinical variables** In Responders, widespread reduction in sgACC FC: Seed-to-whole-brain FC: Reduced FC with left IPL and left OFC Seed-to-network FC: Reduced FC with DMN, FPN and AN In Responders, reduction in FC between bilateral amygdalae and AN In Responders, lower baseline FC between PCC and right anterior INS and right IFG In Responders, negative baseline connectivity between and right anterior INS and right IFG (positive in Non-responders)
Zheng *et al*. (2020)	**Sham-controlled:** No **Blinded:** NA **Control population:** Yes **Psychometric assessments:** HAMD17, HAMA at baseline and 2 weeks fup **Neuroimaging assessment:** MRI at baseline and 2 weeks fup	**Pts**: 27 (41.22 ± 12.71 years, 8M/19F) **HC**: 27 (41.00 ± 8.04 years, M/F 6/21) **Diagnosis**: MDD (DSM-IV) **Severity:** HAMD 23.89 ± 4.47 **Treatment resistance status: NA** **Medication status:** Previously medicated for less than a week. Washout from all medication 1 month before **Psychiatric comorbidities:** Excluded if psychotic disorder, SUD, alcohol abuse	**Region:** Left DLPFC **Neuronavigation:** No **Intensity (% MT):** 100% **Frequency:** 10 Hz **Protocol:** *Train* = 4 s; intertrain interval 26 s *Session* = 50 trains *Total stimuli per session*: 1500 **Duration:** 5 days/week, 2 weeks	**MRI (3T)** **Resting state** **FC method:** ALFF, FCD **Regions:** CEN, DMN and SN	**Baseline differences** Two-sample *t*-tests for differences in FC between pts and HC **Post-TMS changes** Paired *t*-tests for differences in FC between pre- and post-rTMS **Relationship with clinical variables** Pearson correlation between ALFF/FCD and symptom scores at baseline Pearson correlation between ALFF/FCD and symptom scores changes	**Baseline differences** In pts *v.* HC: ALFF increased in right OFC and decreased in left striatal cortex and medial PFC. No differences in ALFF in DMN, CEN and SN FCD increased in right dACC and OFC and decreased in right IPL. FCD decreased in CEN **Post-TMS changes** ALFF increased in left DLPFC and SFG. No differences in ALFF in CEN, DMN or SN FCD increased in right dACC and STG, decreased in bilateral LG. FCD increased in CEN **Relationship with clinical variables** At baseline, FCD in CEN negatively correlated with HAM-A No correlations between changes in ALFF or FCD and changes in symptoms scores after rTMS

99mTc-ECD, ^99m^Tc-ethyl cysteinate dimer; AI, anterior insula; ALFF, amplitude of low frequency fluctuation; AN, Affective Network; ATHF, antidepressant treatment history form; BD, bipolar disorder; BDI, Beck Depression Inventory; CEN, Central Executive Network; DLPFC, dorsolateral prefrontal cortex; DMN, Default Mode Network; DSM, Diagnostic and Statistical Manual of Mental Disorders; DTI, diffusion tensor imaging; EC, effective connectivity; ED, eating disorder; FC, functional connectivity; FCD, functional connection density; FD, framewise displacement; fMRI, functional magnetic resonance imaging; fup, follow-up; GAD7, General Anxiety Disorder, 7 items version; GAF, Global Assessment of Functioning; GC, Granger causality; GM, grey matter; HAMA, Hamilton Anxiety Rating Scale; HAMD17/24, Hamilton Depression Rating Scale (17/24 items version); HC, healthy controls; ICA, independent component analysis; IFG, inferior frontal gyrus; INS, insula; IPL, inferior parietal lobule; iTBS, intermittent theta burst stimulation; LG, lingual gyrus; MADRS, Montgomery-Åsberg Depression Rating Scale; MDD, major depressive disorder; MFG, medial frontal gyrus; MSM, Maudsley Staging Method; MT, motor threshold; OCD, obsessive compulsive disorder; OFC, orbitofrontal cortex; PCC, posterior cingulate cortex; pgACC, pregenual anterior cingulate cortex; preCUN, precuneus; Pts, patients; PTSD, post-traumatic stress disorder; QIDS, Quick Inventory of Depressive Symptomatology; rACC, rostral anterior cingulate cortex; rTMS, repetitive transcranial magnetic stimulation; SCT, Stroop Color Test; SFG, superior frontal gyrus; sgACC, subgenual anterior cingulate cortex; SN, Salience Network; SPECT, single-photon emission computed tomography; SSRI/SNRI, selective serotonin/norepinephrine reuptake inhibitors; STAI-Y, State-Trait Anxiety Inventory, Y version; STG, superior temporal gyrus; SUD, substance use disorder; TCA, tricyclic antidepressants; vmPFC, ventromedial prefrontal cortex; FA, fractional anisotropy; WCST, Wisconsin Cart Sorting Test; WFT, Word Fluency Test; WSAS, Work and Social Adjustment Scale.
